# The Emigrant's Hospital at Ward Island

**Published:** 1889-12-14

**Authors:** 


### THE EMIGRANTS' HOSPITAL AT WARD ISLAND.

WHILE we in England are simultaneously advising our own people to go abroad where they can earn a better living, and crying out against German Jews who have had that same advice given to them in their own country, and have taken it and come here, we hardly realise what a really important business European emigration—which in America becomes immigration—is in New York. The report of the Commissioners of Emigration, annually transmitted to the American Legislature, is a book for which we have no parallel here, though it might be greatly for our advantage if we had, and could say every year how many strangers came to our shores, and what were their nationalities, occupations, and destinations.

A very interesting feature of the system is the Emigrants' Hospital on Ward Island. It is a haven of refuge of the highest order, where the sick arrivals are sheltered till they are fit to find a place for themselves in the new country. But not less is it a desirable precaution for the Americans themselves. Could anything be worse than that some poor alien, ignorant perhaps of the English tongue, and, therefore, unable even to warn those around him of his condition—someone afflicted with infectious disease, should be taken to a common lodging-house, or perhaps allowed to travel by rail, endangering the safety of all with whom he comes in contact. The medical examination of immigrants is a most necessary precaution, and the hospital provided for them a most necessary boon.

The report of Dr. Allen Thomas, the physician-in-chief to the Ward Island Hospital, shows that during 1888 the number of patients cared for was 2,600, distributed among five departments—medical, 1,215; surgical, 413; children's, 167; obstetric, 285; and quarantine, 520. The mortality rate of the institution was 3·21 per cent., or exclusive of cases which died within twenty-four hours of admission, 2·9 per cent. It is needless to point out that the mortality among the sick and friendless poor in common lodging-houses would be much in advance of this. The obstetric department, on which Dr. Thomas dwells particularly, and which has lately been thoroughly renovated, is one of special value, and how carefully the patients are treated there is shown by the fact that in five years only three deaths have occurred, one of which was due to consumption from which the patient had suffered long before admission, while another was suffering from acute Bright's disease when she entered the department. The hospital is not yet perfect, at least it does not yet satisfy Dr. Thomas's high ideal; and, indeed, where a nurse's home, entirely separated from the hospital, does not yet exist, there is still something to be desired. Dr. Thomas also asks for a hospital steamer with proper bunks for the patients and isolated compartments for infectious cases, and he is not wholly satisfied with the sanitary arrangements throughout the institution; but take it for all in all, allowing for every defect, we may well wish that a replica of the Ward Island Hospital were to be found in the port of London.

